# Supplementary material for: Characterization of the WRKY Gene Family Related to Anthocyanin Biosynthesis and the Regulation Mechanism under Drought Stress and Methyl Jasmonate Treatment in Lycoris radiata
Source: Int J Mol Sci. 2023 Jan 26;24(3):2423. doi: 10.3390/ijms24032423 (PMC9917153; doi:10.3390/ijms24032423)

Figure S1 . Conservation and diversity of the motifs in LrWRKY proteins. The schematic representation of ten motifs in WRKY family is elucidated by MEME.

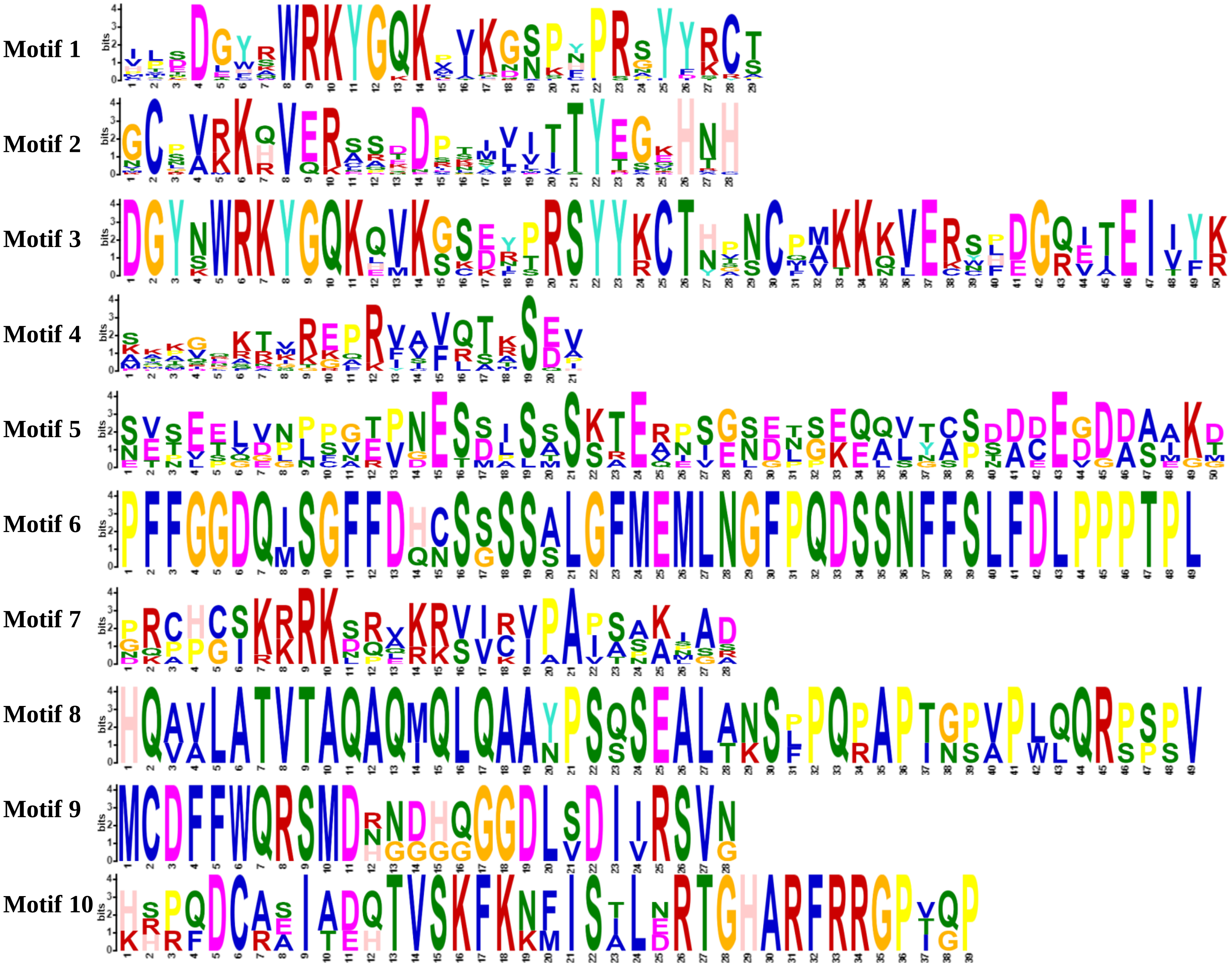

Figure S2 . Expression profile heatmap with hierarchal clustering of *LrWRKYs* in different tissues of *L. longituba*. Red and blue represent high and low relative transcript abundance, respectively.

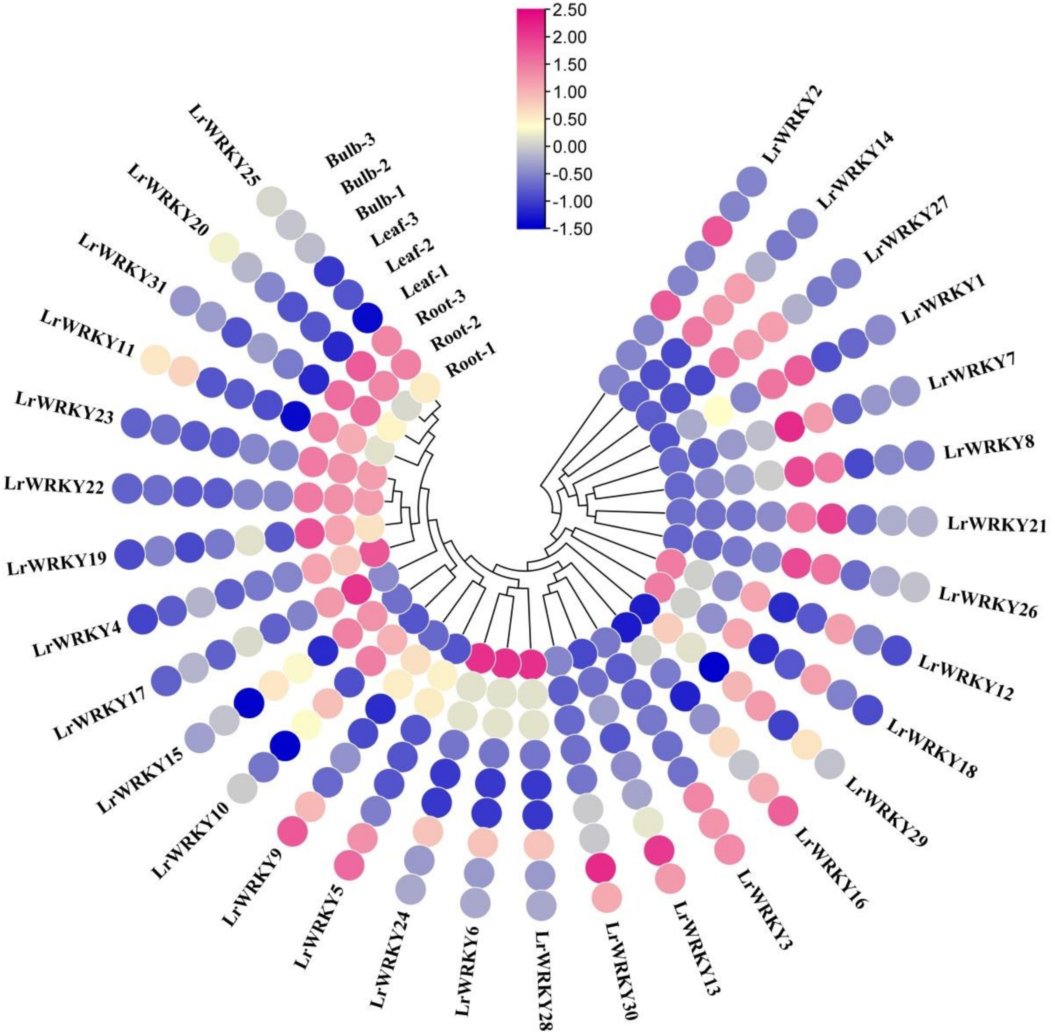

Figure S3 . Heatmap of *LrWRKY* genes expression profiles with MeJA treatment. Red and blue represent high and low relative transcript abundance, respectively.

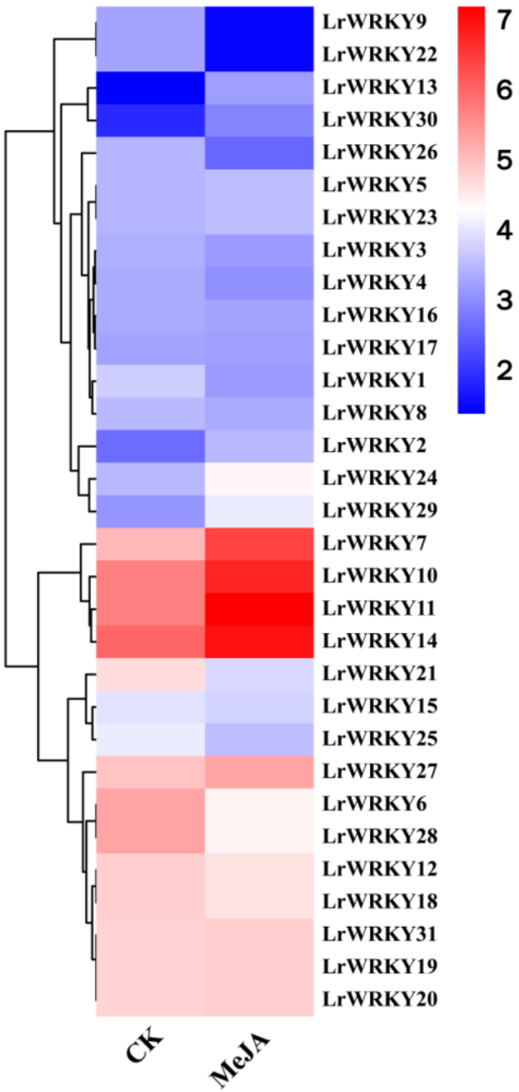

Supplement: Supplementary file 1 [file ijms-24-02423-s001.zip › Supplementary Figure.pdf]
